# Clinically Relevant Genes and Proteins Modulated by Tocotrienols in Human Colon Cancer Cell Lines: Systematic Scoping Review

**DOI:** 10.3390/nu13114056

**Published:** 2021-11-12

**Authors:** Ali Qusay Khalid, Saatheeyavaane Bhuvanendran, Kasthuri Bai Magalingam, Premdass Ramdas, Mangala Kumari, Ammu Kutty Radhakrishnan

**Affiliations:** 1 Jeffery Cheah School of Medicine and Health Sciences, Monash University Malaysia, Bandar Sunway 47500, Malaysia; ali.khalid@monash.edu (A.Q.K.); kasthuri.bai@monash.edu (K.B.M.); 2 Brain Research Institute Monash Sunway (BRIMS), Jeffrey Cheah School of Medicine and Health Sciences, Monash University Malaysia, Jalan Lagoon Selatan, Bandar Sunway 47500, Malaysia; bsaatheeyavaane.bhuvanendranpillai@monash.edu; 3 Division of Applied Biomedical Sciences and Biotechnology, School of Health Sciences, International Medical University, Kuala Lumpur 57000, Malaysia; premdass_ramdas@imu.edu.my; 4 Department of Anatomy, Division of Human Biology, School of Medicine, International Medical University, Kuala Lumpur 57000, Malaysia; mangala_kumari@imu.edu.my

**Keywords:** vitamin E, tocotrienols, colorectal cancer, cell lines, biomarkers, Cytoscape, PRISMA, KEGG, STRING

## Abstract

The last decade has witnessed tremendous growth in tocotrienols (T3s) research, especially in the field of oncology, owing to potent anticancer property. Among the many types of cancers, colorectal cancer (CRC) is growing to become a serious global health threat to humans. Chemoprevention strategies in recent days are open to exploring alternative interventions to inhibit or delay carcinogenesis, especially with the use of bioactive natural compounds, such as tocotrienols. This scoping review aims to distil the large bodies of literature from various databases to identify the genes and their encoded modulations by tocotrienols and to explicate important mechanisms via which T3s combat CRC. For this scoping review, research papers published from 2010 to early 2021 related to T3s and human CRC cells were reviewed in compliance with the PRISMA guidelines. The study included research articles published in English, searchable on four literature databases (Ovid MEDLINE, PubMed, Scopus, and Embase) that reported differential expression of genes and proteins in human CRC cell lines following exposure to T3s. A total of 12 articles that fulfilled the inclusion and exclusion criteria of the study were short-listed for data extraction and analysis. The results from the analysis of these 12 articles showed that T3s, especially its γ and δ analogues, modulated the expression of 16 genes and their encoded proteins that are associated with several important CRC pathways (apoptosis, transcriptional dysregulation in cancer, and cancer progression). Further studies and validation work are required to scrutinize the specific role of T3s on these genes and proteins and to propose the use of T3s to develop adjuvant or multi-targeted therapy for CRC.

## 1. Introduction

Colorectal cancer (CRC) is the world’s third most prevalent malignancy and the fourth leading cause of cancer mortality, with almost 1.4 million new cases and approximately 700,000 deaths reported annually [1]. Considering its prevalence, by 2030, the CRC burden is projected to rise by 60%, to over 2.2 million new cases and 1.1 million deaths [2]. More than two thirds of all patients and about 60% of CRC-related deaths are found in countries with a high or extremely high human development index (HDI) [1,2].

**Figure 1:**
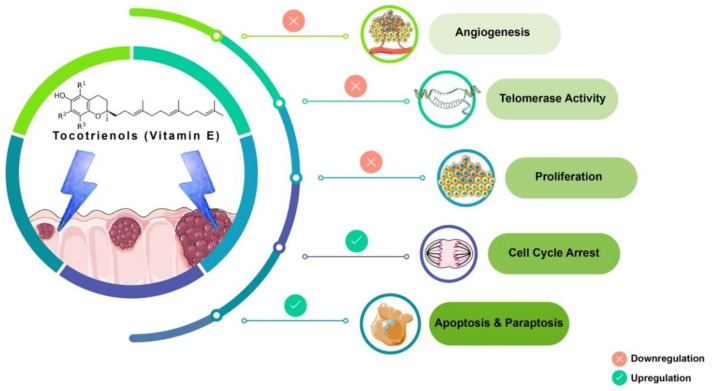
Anticancer actions of tocotrienols. Tocotrienols have been reported to exert anticancer effects by several mechanisms, such as induction of cell death via apoptosis and paraptosis in cancer cells, cell cycle arrest, inhibition of proliferation, suppression of the expression of the human telomerase reverse transcriptase (hTERT) in cancer cells as well as inhibition of angiogenesis. [α-tocotrienol: R1 = Me, R2 = Me, R3 = Me; β-tocotrienol: R1 = Me, R2 = H, R3 = Me; γ-tocotrienol: R1 = H, R2 = Me, R3 = Me; δ-tocotrienol: R1 = H, R2 = H, R3 = Me.

Tocotrienols (T3s), members of the vitamin E family, are natural compounds found in various food sources and exist as four naturally occurring analogues known as alpha (α), beta (β), delta (δ), and gamma (γ). The general chemical structure of tocotrienols is shown in Figure 1. Each analogue has different functional groups that are denoted by R1, R2, and R3 [3]. In α-tocotrienol, R1, R2, and R3 represent methyl (Me) groups, but in β-tocotrienol, R1, R2, and R3 represent Me, hydrogen (H), and Me groups, respectively. In γ-tocotrienol, R1 represents H while R2 and R3 represent Me groups. However, in δ-tocotrienol, R1 and R2 represent the H group while R3 stands for the Me group [3]. The T3s are unsaturated and possess an isoprenoid side-chain, which allows them to efficiently penetrate tissues with saturated fatty layers. The anticancer potential of T3s is only beginning to receive recognition as several mechanistic studies have shown that T3s have unique anticancer properties, which are modulated through several cancer-associated mechanisms and pathways (Figure 1) [3,4], such as inhibition of telomerase activity through suppression of protein kinase C (PKC) activity in cancer cells [5]; and blocked expression of hypoxia-induced vascular endothelial growth factor (VEGF), interleukin-8 (IL-8), and cyclooxygenase 2 (COX-2), which play critical roles in cancers as these act as autocrine growth factors for carcinogenesis and tumor neovascularization [6,7,8]. On the contrary, T3s are reported to enhance the expression of p21 and p27, both known to cause cell cycle arrest [9]. In addition, T3s can induce apoptosis in cancer cells through several mechanisms, which includes inhibition of the nuclear factor-κB (NF-κB) pathway and its regulated gene products [10] as well as regulation of both intrinsic and extrinsic apoptotic pathways by modulating the caspase cascades, expression of B cell lymphoma 2 (BCL2), BCL2-associated X protein (BAX), and tumor necrosis factor-related apoptosis-inducing ligand (TRAIL) by upregulating the expression of death receptors (DRs) [11,12].

Tocotrienol is a form of vitamin E that is gaining recognition for its immense benefits against various types of cancers and other diseases. The anticancer effects of T3s shown in Figure 1 only represent a small portion of the overall T3-based research. The current state of information on this lesser-known type of vitamin E calls for more investigations to garner more scientific evidence that could further strengthen T3s’ many functions and attributes in various experimental models.

This systematic scoping review is aimed at identifying clinically relevant genes and proteins regulated by T3s in human CRC cell lines from research articles published in the last 10 years. Using this approach, the molecular interactions of T3-regulated genes and proteins were evaluated to further understand their functional role in pathways associated with CRC. Besides, the evidence presented in this scoping review will further uncover novel information related to T3 and its potential as an anti-CRC agent.

## 2. Materials and Methods

### 2.1. Search Strategy

For the literature search, we followed the Arksey and Malley Scoping Studies Methodological Framework [13] and PRISMA Statement [14] to design, analyze, and report data throughout this scoping review. We looked for “exemplar studies” to identify relevant search terms on “tocotrienols (T3s)” and “colon cancer cell lines”. A combination of titles, keywords, and subject headings were used for the following conceptual PICO question [15]. Whereas P (problem) was identified as human colorectal/colon cancer cell lines, I (intervention) was the various analogues of T3s used for testing; C (comparison) was the outcomes compared with untreated cells; and O (outcomes) was the genes or proteins that were differentially expressed in these colon cancer cells treated with T3s. A systematic search of research articles was performed to screen for the genes or proteins differentially expressed in human CRC cell lines following treatment with various T3s analogues published in English. The search was conducted in four databases, while the key terms that formulated the search query were tocotrienols, genes, proteins, colon cancer, and colorectal cancer.

### 2.2. Search Method

A list of terms with a similar concept were searched in the databases. In Ovid MEDLINE, PubMed, Scopus, and Embase, the search was done for the general query “TITLE-ABS-KEY (((tocotrienol*) AND ((gene*) OR (protein*)) AND ((colon) OR (colorectal)) AND (cancer))) AND (LIMIT-TO (PUBYEAR, 2021) OR LIMIT-TO (PUBYEAR, 2020) OR LIMIT-TO (PUBYEAR, 2019) OR LIMIT-TO (PUBYEAR, 2018) OR LIMIT-TO (PUBYEAR, 2017) OR LIMIT-TO (PUBYEAR, 2016) OR LIMIT-TO (PUBYEAR, 2015) OR LIMIT-TO (PUBYEAR, 2014) OR LIMIT-TO (PUBYEAR, 2013) OR LIMIT-TO (PUBYEAR, 2012) OR LIMIT-TO (PUBYEAR, 2011) OR LIMIT-TO (PUBYEAR, 2010)) AND (LIMIT-TO (DOCTYPE, “Research Articles”)) AND (LIMIT-TO (LANGUAGE, “English”)).”

Variations of search queries were used based on the structure of databases. The literature found through the searches was exported into Endnote X9 literature management software to remove duplicates. Following this, the papers were imported into Covidence (https://www.covidence.org/), which was last accessed on 15 May 2021, where two independent researchers screened the title and abstract of these papers to identify related studies. Conflicts were resolved by a third researcher, who was blinded to the initial screen. Articles that did not fulfil the selection criterion were eliminated. Following this, the full texts of these articles were further assessed based on inclusion and exclusion criteria. Data charting was done independently by two reviewers. Reviewers assessed the vital information to be analyzed and the articles to be included for analysis. A standardized data charting template developed with Microsoft Excel was used by both reviewers (Table 1).

### 2.3. Criteria to Select Studies

All studies on human colon cancer cell lines that reported gene or protein regulation following treatment with T3s analogues were selected in accordance with the inclusion criteria of (i) published within the study period (January 2010 to May 2021), (ii) published in English, and (iii) full text of the paper was available. Animal studies, non-human colon cell lines, human trials, reviews, non-English articles, and conference abstracts were excluded. In addition, we did not use any manual adding methods to avoid selection bias. The criteria applied for the search are provided in the Supplementary Materials.

### 2.4. Functional Bioinformatics Analysis

The genes and proteins that were reported in at least two out of the 12 research articles (Table 1) were selected and analyzed using four bioinformatics tools: (i) Venn Diagram Analysis, (ii) Search Tool for the Retrieval of Interacting Genes (STRING), (iii) Cytoscape Molecular Network, and (iv) Kyoto Encyclopedia of Genes and Genomes (KEGG).

#### 2.4.1. Venn Diagram Analysis

The differentially expressed genes and proteins were analyzed using the Venn diagram [28]. The analysis allowed identification of unique and overlapped proteins across various CRC cell lines (HT-29, HCT-116, DLD-1, and SW620), following exposure to γT3 and δT3.

#### 2.4.2. STRING Interaction Analysis

The STRING database and its online resources [29] were used to perform functional classification analysis of genes and proteins that were reported in at least two out of the 12 research articles (Table 1) to determine the functional connections between differentially expressed proteins based on direct (physical) and indirect (functional) associations. This was performed by uploading the “UniProt” IDs of the proteins corresponding to the genes identified into the multiple protein analysis interface in STRING and selecting “Homo sapiens” to investigate the protein–protein interactions (PPIs). Evidence, confidence, and molecular action network edges were used to evaluate PPIs. The interaction score was set to high confidence (0.700) to populate protein groups with similar correlations, and K-mean clustering was used in the analysis.

#### 2.4.3. Cytoscape Molecular Network Analysis

The PPI file was downloaded from STRING in TSV, where it can be directly uploaded into Cytoscape software (Version: 3.8.2/Java: 11.0.6) [30]. It is an open-access platform for molecular complex network construction and visualization, combined with annotations, gene expression status, and data sources. A 3D-rational model was developed by arranging the proteins according to their expression level. The ellipse shape represents upregulation, and the hexagon shape represents downregulation, while the octagon shape represents a contradiction.

#### 2.4.4. KEGG Pathway Analysis

A Kyoto Encyclopedia of Genes and Genomes (KEGG) analysis was conducted to determine the potential proteogenomic functional annotation and pathway enrichment association. KEGG is a collection of databases that connect genomic and high-level functional data. The acknowledged figure was adopted from the KEGG database under the pathway entry code: hsa05210 (Colorectal cancer—Homo sapiens). Only proteins with high engagements, identified by Cytoscape, were subjected to KEGG analysis by dividing them into two categories (downregulated group and upregulated group).

## 3. Results

### 3.1. Selection of Articles

**Figure 2:**
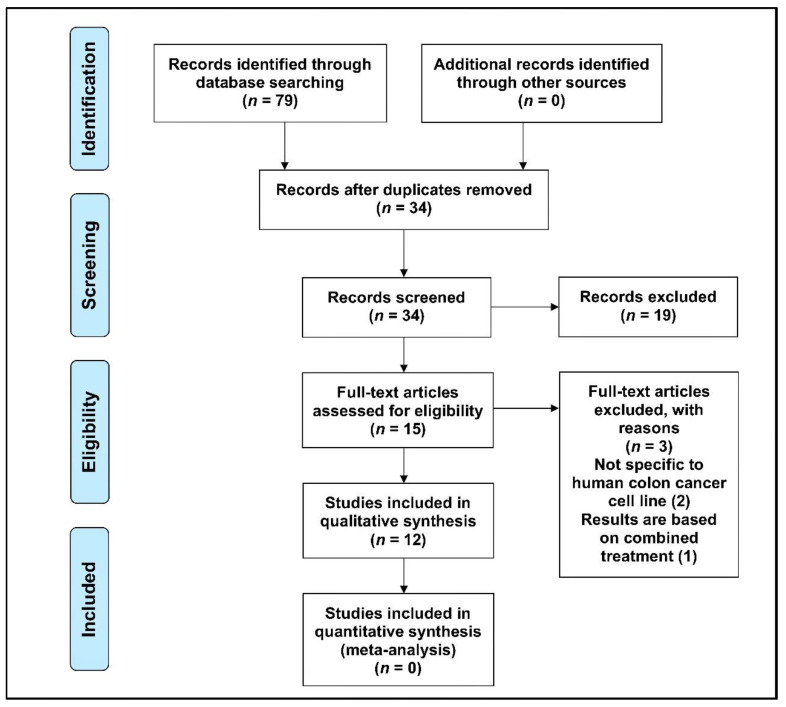
Research articles were searched on four databases (Ovid MEDLINE, PubMed, Scopus, and Embase) that reported using the preferred reporting items for systematic reviews (PRISMA) flow chart.

The original search yielded 79 studies. After removing duplicate papers and screening for inclusion and exclusion criteria, 34 articles were independently read/reviewed by three authors (AQK, SB, and AKR). The relevance of the abstracts was checked. Only research papers that met the inclusion and exclusion criteria of this study, i.e., investigated expression of gene(s) or protein(s) in human CRC cell lines following treatment with T3s, were selected for data extraction and analysis. Research papers that did not meet the inclusion and exclusion criteria of this study (19 articles) were removed from this study. Following this, the remaining articles (15 articles) were thoroughly checked and included for data extraction. During the data extraction process, three articles had to be excluded as they were not directly related to the objectives of this study. Finally, only 12 articles that fit this study’s inclusion and exclusion criteria were used for data extraction and analysis (Figure 2).

The data charting of genes and proteins (proteogenomic) modulated in human CRC cell lines following treatment with various forms of T3s is summarized in Table 1. From the 12 studies, 37 proteins and 9 genes that were differentially expressed in human CRC cell lines were identified. The studies exhibited variance in the findings due to different interventions, cell lines, controls, and techniques. Out of the four analogues of T3s, only two (γT3 and δT3) were used in the majority of the 12 short-listed papers and these analogues (γT3 and δT3) were reported to have higher anticancer activity compared to other T3s analogues or alpha-tocopherol (αToc) [31,32]. A new formulation known as the gamma-delta tocotrienol (GDT) (75:25) was found to have increased anticancer effects compared to the currently available tocotrienol-rich fraction (TRF) formulation, which contains α, δ, and γ tocotrienols in addition to αToc [33].

### 3.2. Human Colon Cancer Cell Lines Studied

**Figure 3:**
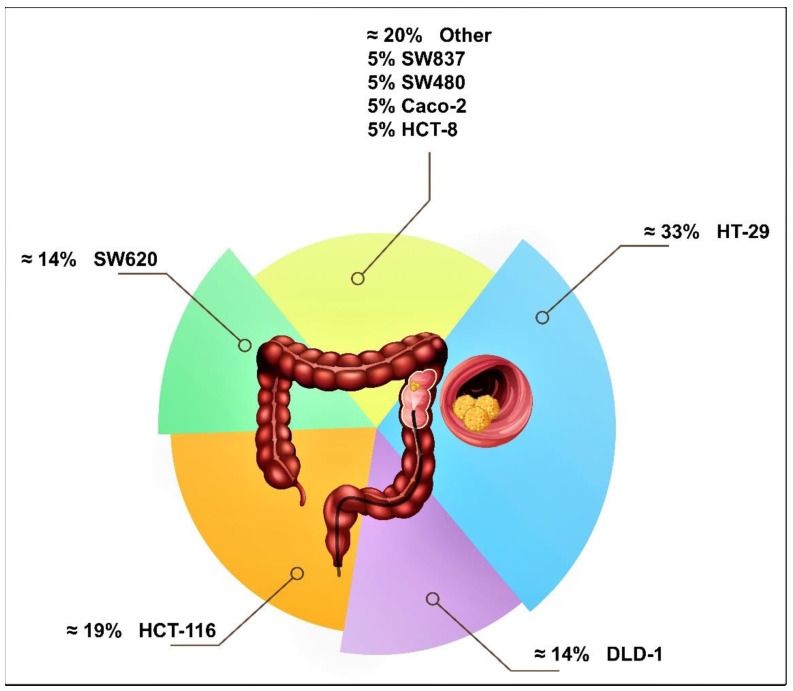
Pie chart showing the relative distribution of the commonly used human colorectal carcinoma (CRC) cell lines as a cell-based model of CRC. These eight human CRC cell lines represent most of the human CRC cell lines used in the 12 research articles selected for this scoping review. Micrographs (Scale bar: 100 μm).

The selection of the cell line in many studies was based on genomic studies, which showed that human CRC cell lines in general mimic the genetic modifications and pharmacogenomics of primary CRC tumors [34]. To date, there are about 54 types of CRC cell lines [35], but in the 12 short-listed articles, only eight of the most common types of human CRC cell lines were used (Figure 3). The most commonly employed human CRC cell lines were HT-29, HCT-116, DLD-1, and SW620 (Table 1). However, the most commonly used cell line was HT-29, which was identified as a useful model for CRC therapeutic testing [36,37,38], possibly due to its sensitivity to standard treatment controls.

### 3.3. Identification of Candidate Biomarkers in the Human Colon Cancer Cell Lines

**Figure 4:**
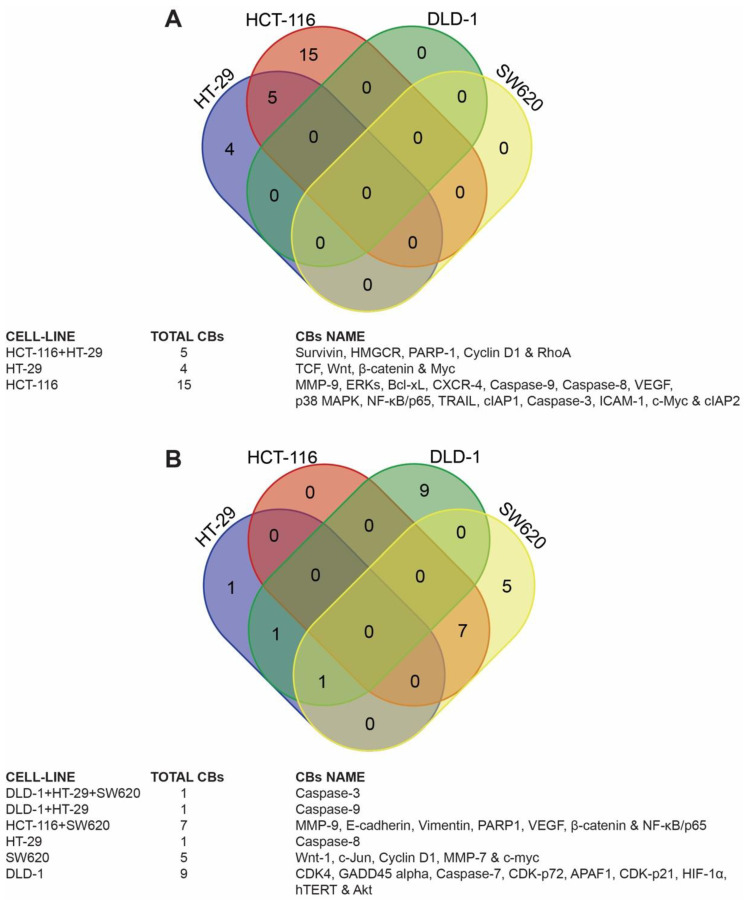
Venn diagrams illustrating the unique candidate genes and proteins present in the cell lines with the highest research usage (HT-29, HCT-116, DLD-1, and SW620) following exposure to T3 analogues. (**A**) Gamma-tocotrienol (γT3); (**B**) Delta-tocotrienol (δT3).

The reported proteogenomic data in the extracted research articles (Table 1) were selected for further analysis in CRC cell lines with the highest research percentages (HT-29, HCT-116, DLD-1, and SW620) following treatment with γT3 and δT3 analogues. The Venn diagram is shown in Figure 4, which reports some key findings.

### 3.4. Target Biomarkers

**Figure 5:**
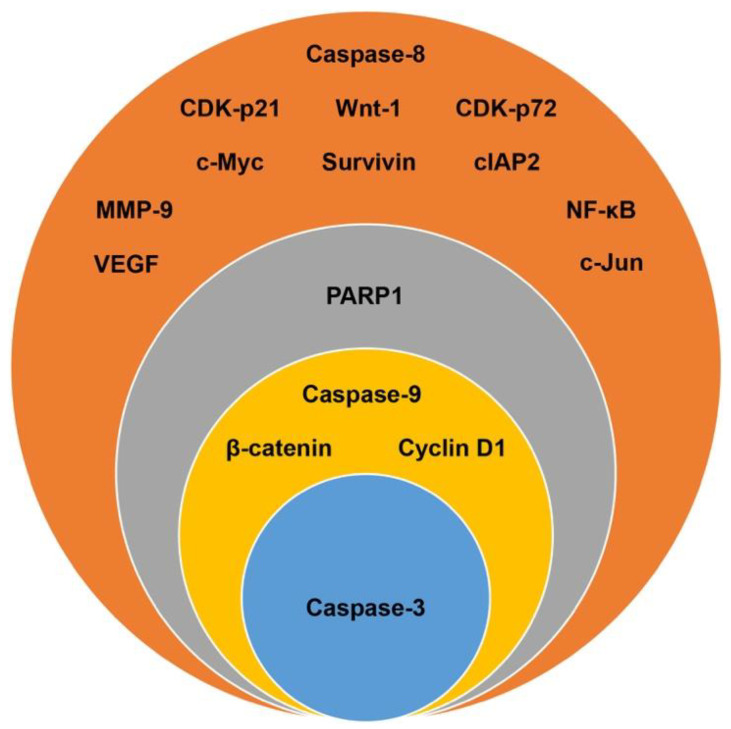
Replicability of 16 candidate biomarkers (CBs) identified in the 12 independent research papers selected for this study. The number of papers that each CB is reported in is arranged in descending order starting with the larger outer circle (orange), which shows CBs reported in two studies, followed by the grey circle, which shows CBs reported in three research papers; yellow circle showing CBs reported in four research papers, and finally the blue circle that shows the CBs reported in five research papers. The CBs reported in a single research paper were excluded.

A total of 37 candidate biomarkers (genes and proteins) were reported in the 12 short-listed studies (Table 1). Among these candidate biomarkers, 16 that were reported in two or more research articles were selected for further analysis. Only caspase-3 was reported in five independent studies [16,18,21,22,24] while caspase-9 [16,18,22,24], beta-catenin (β-catenin) [19,20,21,27], and cyclin D1 [19,20,21,26] were reported in four different studies (Figure 5). Poly-ADP-ribose polymerase 1 (PARP-1) was reported in three studies [17,18,27]. The remaining 11 candidates (68%) were reported in two of the 12 studies and these include caspase-8 [18,24], cyclin-dependent kinase inhibitor 1 (CDK-p21) [16,22], cyclin-dependent kinase inhibitor 1B (CDK-p72) [16,22], proto-oncogene wingless-related integration site 1 (Wnt-1) [19,21], baculoviral IAP repeat-containing protein 3 (cIAP2) [18,26], matrix metallopeptidase 9 (MMP-9) [26,27], vascular endothelial growth factor (VEGF) [26,27], nuclear factor kappa-light-chain-enhancer of activated B cells (NF-κB) [26,27], survivin [20,26], c-Myc [19,26], and c-Jun [19,21] (Figure 5). The remaining 21 candidate genes or proteins, which were reported in only one research paper, were not selected for further analysis.

### 3.5. Differentially Expressed Candidate Proteins and Their Interactions

**Figure 6:**
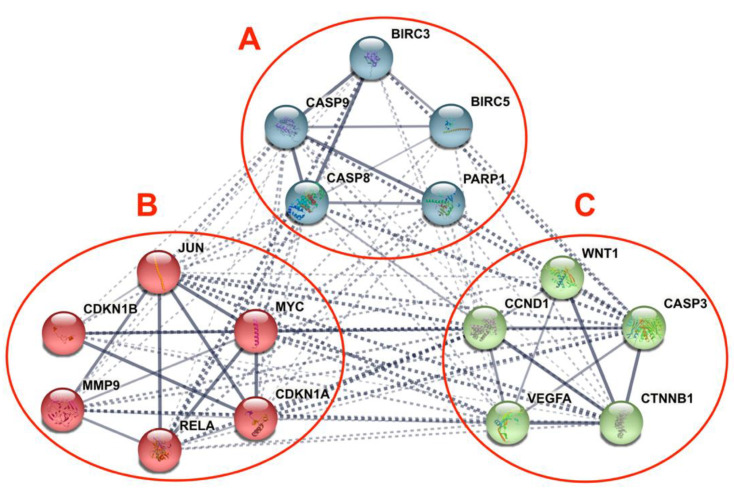
The interaction network of differentially expressed candidate biomarkers (CBs) in human CRC cell lines treated with γT3 or δT3 using STRING, an online bioinformatics software (STRING), which shows protein clusters with high confidence interactions between associated nodes (line thickness indicates the strength of data support). (**A**) CBs involved in apoptosis; (**B**) CBs involved transcriptional dysregulation in cancer; and (**C**) CBs reported being involved in cancer progression pathways.

The PPI of the differentially expressed genes and proteins in the human CRC cell lines in response to γT3 or δT3 generated a total of 97 edges, among which 24 edges were of high confidence, which generated significant (*p*-value < 1.0^−16^) PPI enrichment clusters with a local clustering coefficient of 0.881 and an average node degree of 12.1 (Figure 6). In addition, a total of three clusters were generated using the K-means clustering tool in the STRING software (Figure 6). The three clusters generated contained genes or proteins involved in apoptosis (BIRC3, BIRC5, PARP1, CASP8, and CASP9), transcriptional dysregulation in cancer (CDKN1B, RELA, CDKN1A, MYC, JUN, and MMP9), and cancer progression (CCND1, CASP3, CTNNB1, WNT1, and VEGFA) pathways.

## 4. Discussion

Inherited CRC is now acknowledged as a significant factor in the development of this disease. Genome sequencing-based diagnosis estimates that one-third of CRC patients appear to have familial colorectal cancer (FCC) as a part of their pathogenesis [39]. The initiation of CRC arises from the epithelial tissue due to the accumulation of genetic modulations in specific oncogenes and tumor suppressor genes (TSGs). In the evolution of sporadic CRC, two primary mechanisms of genomic instability have been identified. The first is chromosomal instability (CIN) caused by a cascade of genomic alterations involving activating oncogenes like *KRAS* and the inactivation of TSG like *p53*, *DCC*/*SMAD4*, and *APC*. The second, known as microsatellite instability (MSI), is caused by hypermethylation of the promoters of the DNA mismatch repair genes *MLH1* and/or *MSH2*, as well as secondary mutation of genes with coding microsatellites, such as transforming growth factor receptor II (*TGF-RII*) and *BAX* [40,41,42]. In comparison, germline mutations in the defined genes may lead to inherited CRC. Those mutations can be in the tumor suppressor gene *APC* on chromosome 5q as in familial adenomatous polyposis (FAP) or mutated DNA mismatch repair genes in hereditary non-polyposis colorectal cancer (HNPCC) [43,44]. In this systematic scoping review, differentially expressed candidate proteins in response to γT3 or δT3 treatment were retrieved to identify the molecular mechanisms through which these T3s isoforms modulate anticancer effects.

The 16 CBs (BIRC3, BIRC5, CASP3, CASP8, CASP9, CCND1, CDKN1A, CDKN1B, CTNNB1, JUN, MMP9, MYC, PARP1, RELA, VEGFA, and WNT1) short-listed in this study formed three prominent clusters that were part of the apoptotic, transcriptional misregulation, or cancer progression pathways (Figure 6). All three pathways are interlinked and are crucial with respect to anticancer mechanisms. Hence, it is highly likely that the 16 CBs play a pivotal role in mediating anticancer mechanisms induced in human CRC cell lines exposed to γT3 or δT3. A number of the CBs have been reported to play important roles clinically in the carcinogenesis of patients with CRC (Table 2).

**Figure 7:**
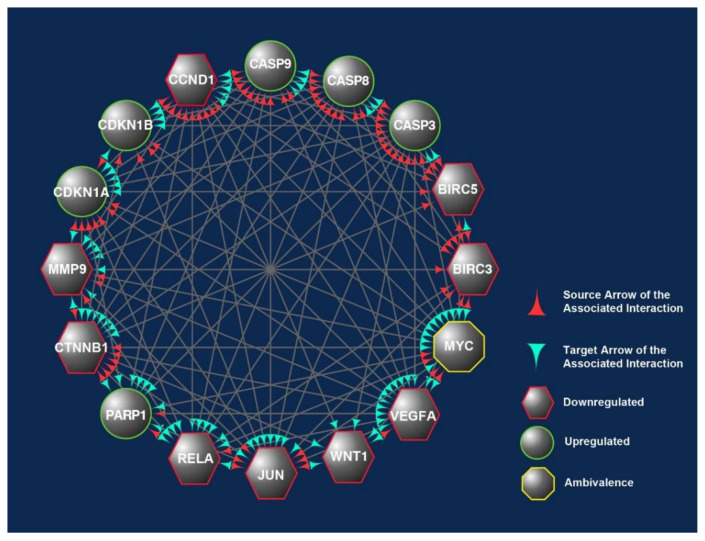
The overlapped targets of the 16 candidate biomarkers (CBs) identified in this study were populated according to their engagements using Cytoscape, an online bioinformatics tool. The protein–protein interactions (PPIs) between these CBs formed a prominent network. The CBs in the hexagons and circles are significantly downregulated or upregulated in human CRC exposed to γT3 or δT3. In contrast, the CB in the octagons is a biomarker that did not show significant changes. The green and red arrowheads reflect the interactions between these CBs. The red arrowheads indicate source CBs protein that can exert stimulatory (circles) or inhibitory (hexagons) or no effects (octagons) on target CBs (green arrowheads).

This was evident when these CBs were analyzed using another bioinformatics software, i.e., Cytoscape, which shows the connections or interactions between these biomarkers (Figure 7). In this analysis, 10 of the CBs (CASP3, CASP8, CASP9, CCND1, CDKN1A, CTNNB1, JUN, MYC, RELA, and VEGFA) were found to play critical roles in the protein–protein interactions (PPIs) as there were correlated interactions between these CBs. We observed between 12 and15 engagements marked with either of these CBs, being the protein that exerts an effect (source) or affected by the CBs’ action (target) (Figure 7) [30].

**Figure 8:**
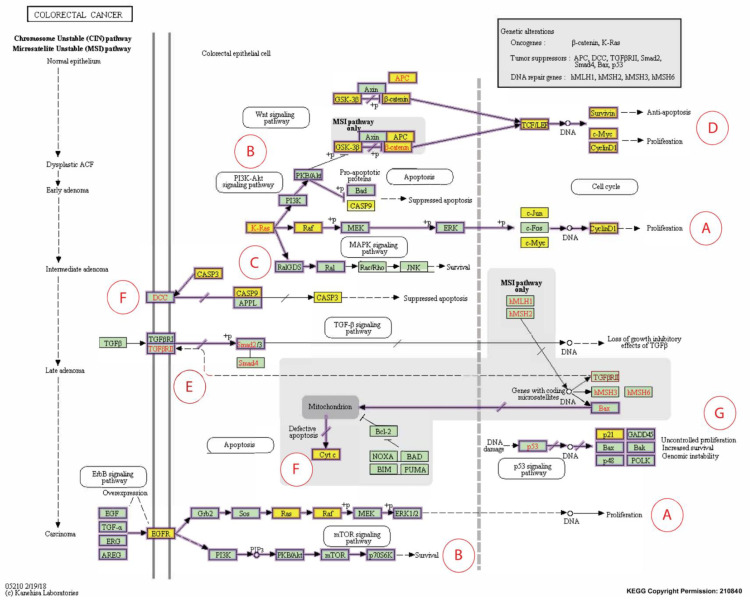

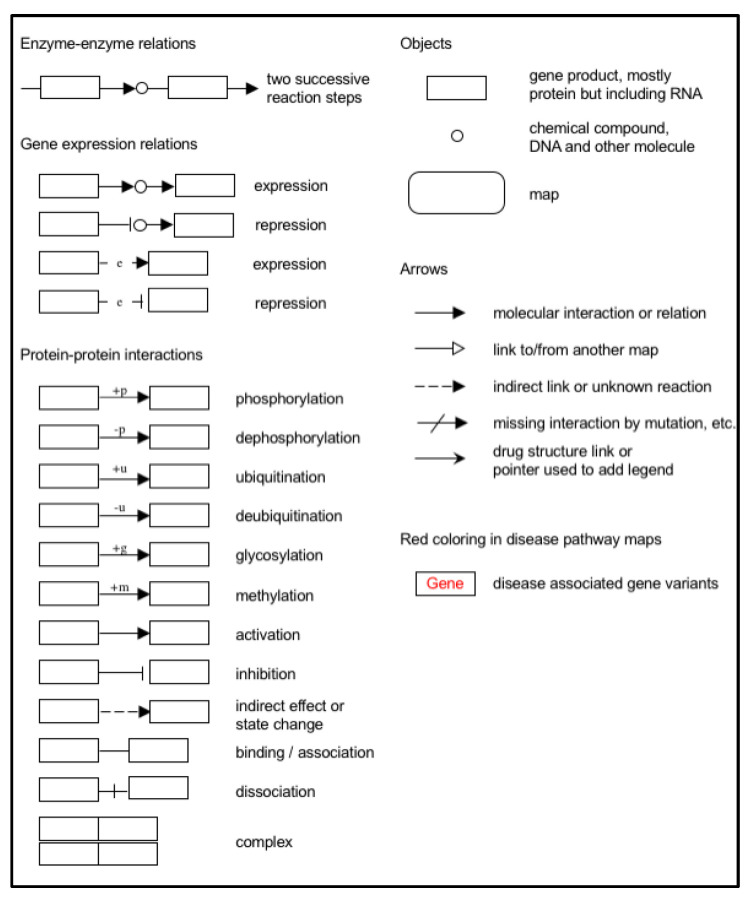
Expanded Kyoto Encyclopedia of Genes and Genomes (KEGG) pathway representation of colorectal carcinoma showing the involvement of the 16 candidate biomarkers selected for this study in the pathogenesis of CRC. The cancer pathways that these 16 CBs are involved in include (**A**) ERK signaling; (**B**) PI3K signaling; (**C**) RAS signaling; (**D**) WNT signaling; (**E**) TGFB signaling; (**F**) Apoptotic signaling; (**G**) Transcription. Genes marked with yellow rectangles represent the 16 CBs identified in this scoping review.

Caspase-3 (CASP3) is the only CB reported in five independent studies as overexpressed following exposure to T3s. So, therapeutic targeting of caspase-3 may boost cancer cell susceptibility to chemotherapy and irradiation while simultaneously inhibiting invasion and metastasis. Using the CRISPR technology, Zhou et al. [57] established a caspase-3 knockout (KO) human CRC cell line where the caspase gene was knocked out in the HCT116 human CRC cell line (CASP3KO). The authors reported that the CASP3KO-HCT116 cells were less clonogenic, less invasive, and more susceptible to mitomycin-C treatment than the wild-type control cells [57]. In addition, the CASP3KO-HCT116 cells proliferated at a similar rate as the control cells in vivo and were more sensitive to radiation. When administered subcutaneously or intravenously, these cells were less prone to pulmonary metastases than the wild-type HCT116 cells. Deletion of the *CASP3* gene also generated lesser EMT phenotypes on a molecular level [57]. In a clinical study, irradiated CRC cells undergoing apoptosis and necrosis were reported to produce significantly higher levels of cleaved caspase-3 (CC3) [45]. The immunohistochemistry staining revealed that the colorectal tumor tissues also showed significantly higher expression of CC3 compared to the peri-tumoral tissues. The authors concluded that high CC3 levels were related to poor prognosis [45]. When the roles of caspase-3 and CC3 were analyzed using the colorectal cancer pathway provided by the KEGG database [58], there was substantial evidence to show that suppression of caspase-3 or increased expression of CC3 suppressed apoptosis, and this could be one of the reasons why this correlates with poor prognosis (Figure 8F).

The *CCND1* gene encodes cyclin D1, a biomarker of interest that was reported to be modulated in human CRC cells following exposure to T3s in four out of the 12 short-listed research papers. For instance, in a clinical study, the *CCND1* gene was detected in tumors from about 50% (54 out of 111) of CRC patients, but the expression of this gene was absent in normal mucosa [54]. When the cyclin D1 protein expression was investigated in these CRC patients, it was found that this protein could be detected in tumor tissues from 69 cases, of which gene expression was detected in 43 [54]. In the same paper, the authors reported a significant relationship between the expression of the *CCND1* gene and protein (cyclin D1). Furthermore, there was a significant link between the expression of the *CCND1* gene and metastasis to lymph nodes or distant tissues. Therefore, the author concluded that combined measurement of the *CCND1* gene and its protein, cyclin D1, is crucial for use as molecular predictors of human CRC [54]. In addition, when the roles of the cyclin D1 protein were analyzed using the cancer pathway (colorectal cancer) provided by the KEGG database [58], there was substantial evidence to show that downregulation of this protein can reduce the cancer burden (Figure 8A,D) [54,58,59].

Vascular endothelial growth factor (VEGF) is a potent angiogenic protein secreted by almost all types of cancers. The VEGF family includes four ligands and three receptors, of which vascular endothelial growth factor A (VEGFA) is the best-known member. In most papers, exposure to T3s caused downregulation of VEGFA in the CRC cells. Furthermore, in a clinical study involving lymph node metastasis (LNM) from patients with CRC, there was elevated expression of the VEGF family, especially of VEGFA in LNM, which was associated with the patients’ age (*p*-value < 0.001) [55]. Interestingly, high expression of VEGFA in the primary tumor was positively associated with other ligands and receptors with regards to LNM, implying a mutual effect. Hence, downregulation VEGFA in cancers can be regarded as a good therapeutic outcome.

The apoptosis pathway plays a significant role in inhibiting the tumorigenic progress. A critical factor in the etiopathology of several cancer therapies, such as chemotherapy and irradiation, is to destroy cancers by triggering apoptosis [60]. There are two main apoptotic pathways, i.e., intrinsic and extrinsic pathways. Activation of caspase-8 (CASP8), a cysteine protease, via engagement of various death receptors initiates the extrinsic apoptotic signaling pathway [61,62] and this induces the release of Cyt c from the mitochondria, inducing cell death by activating caspase nine and other apoptosis mediators (Figure 8F).

Five somatic mutations in the *CASP8* gene were identified in 98 invasive colorectal carcinomas (5.1%) but not in any adenomas [46]. Of these five mutations, one was the result of a frameshift mutation, one was due to a nonsense mutation, and the remaining three were missense mutations. In addition, the prevalence of caspase-8 mutations was substantially higher in carcinomas (*p*-value = 0.05) and there was significant reduction of the apoptotic activity in tumors harboring the caspase-8 mutations [46]. It was proposed that the presence of mutant caspase-8 in colon carcinomas showed that mutations in the *CASP8* gene may have resulted in the loss of its apoptotic function and restoration of this activity may promote tumor apoptosis for the treatment of CRC [47]. The expression of the *caspase-9* (*CASP9*) gene was downregulated in CRC tissue when compared to the corresponding tissue from normal mucosa (*p*-value = 0.001) [48]. In addition, patients with downregulated caspase-9 had a lower overall survival (*p*-value = 0.012) and disease-free survival (*p*-value = 0.022) [63]. Therefore, caspase-9 (*CASP9*) could be a useful biomarker in predicting the prognosis of CRC patients [63] (Figure 8F). Exposure to T3s increased the expression of the *CASP8* and *CASP9* genes in the human CRC cell lines, making it a good target molecule for further studies to evaluate its potential to be used to treat CRC.

CDKN1A (p21) is one of the cyclin-dependent kinase (CDK) inhibitors that is transcriptionally controlled by p53; a transcription factor that plays a vital role in the regulation of the cell cycle. Deletions in the *p21* gene were found in 371 (50%) out of the 737 CRC samples analyzed from two prospective cohort studies [49]. Further analysis showed that mutations in a proto-oncogene (*BRAF* gene) were inversely related to p53 expression and loss of p21 expression. In addition, the correlation between the expression of the *p2*1 gene and mutations in the *BRAF* gene in the CRC tissues was evident when their p53 status was stratified. In contrast, the relationship between p53 positivity and the mutations in the *BRAF* genes was no longer evident in CRC when the p21 status was stratified [49]. The relationship between *BRAF* and *p21* genes can be observed in the KEGG colorectal cancer pathway (Figure 8A–C) [58]. Different cancer prognosis and survival types have been linked to somatic changes in genes that regulate cell division. P21 is a crucial regulator of the cell cycle (Figure 8G) [64].

The *CTNNB1* genes encode β-catenin protein, which support tumor growth. In 80 human CRC tumor specimens stratified by the presence or absence of microsatellite instability (MSI), mutations in the *CTNNB1* gene were found in 53 tumor specimens (25%) with high-frequency MSI (MSI-H) but no mutations were observed in the *CTNNB1* gene in the 27 MSI tumors with low-frequency MSI (MSI-L) [56]. The authors concluded that there was a significant link between *CTNNB1* mutations and MSI [56]. Furthermore, 46% of the *CTNNB1* mutations in endometrial cancer were reported to be immediately phosphorylated by glycogen synthase kinase-3 (GSK-3β). It was proposed that the discrepancies in the mutation profiles show that *CTNNB1* mutations may have molecular fingerprints determined by biological factors, such as tumor type and underlying genomic instability pathways, so-called transcriptional misregulation pathways [65]. The proposed role of β-catenin is shown in the KEGG human colorectal cancer pathway (Figure 8D) [58].

The Jun family genes’ products, such as *c-Jun*, *JunB*, and *JunD*, are crucial components of the activating protein-1 transcription factor complexes, which regulate cell proliferation, differentiation, and neoplastic transformation [50]. Although higher c-Jun expression has been observed in many studies concerning CRC (Figure 8A), the expression of JunB and JunD in these tumors has yet to be investigated. Therefore, Wang and his team looked at the expression of c-Jun, JunB, and JunD proteins in 24 cases of human CRC [50]. In identical colectomy specimens, normal-appearing colonic mucosa far from the tumors was employed as a comparison point. According to the findings, in normal mucosa, both c-Jun and JunB proteins were undetectable or barely detectable, but their expression levels were dramatically enhanced in human colorectal adenocarcinomas. JunD protein, on the other hand, was abundant in normal mucosa and only showed a slight increase in adenocarcinomas. These findings point to the possibility that distinct Jun proteins play diverse roles in colonic epithelial cell proliferation and carcinogenesis. Its downregulation might be key to limiting cancer spread.

Epidermal growth factor receptor (EGFR) is a significant oncogene found in various malignancies [66]. Anti-EGFR resistance in metastatic colorectal cancer (MCRC) may be linked to changes in the transcription factor c-MYC (MYC) (Figure 8A,B,D). The expression of MYC was quantified in 121 MCRC patients who had wild-type *RAS* and *BRAF* genes before and after treatment with a combination of anti-EGFR and Folfiri therapy as well as in 33 subsequent metastases collected during target therapy [51]. When compared to patients with low MYC expression (LME), those with higher MYC expression (HME) had a significantly shorter progression-free survival time (PFS) and overall survival (OS) [51]. Furthermore, after TT, the HME pattern was substantially more common in metastases, related to anti-EGFR molecular resistance changes. Furthermore, expression gene profiling revealed that MYC plays a critical role in CRC-related cell cycle, apoptosis, signaling, and cell growth pathways. Patients with anti-EGFR-treated MCRC who have higher MYC expression may have a shorter PFS and OS. Identifying specific miRNAs involved in regulating the MYC pathway and downstream MYC effector genes may provide a new target for overcoming anti-EGFR resistance in MCRC. Although few studies suggested that MYC overexpression might sensitize CRC cells to induced apoptosis, we generated a contradicting finding in this review about its up- and downregulation [67,68,69].

RELA/p65, a vital element of the NF-κB signaling cascade, has various roles in oncogenesis. Apart from being an essential member of RNA metabolism, RNA helicase p68 also works as a transcriptional coactivator of numerous oncogenic transcription factors, including β-catenin, and has been linked to cancer progression. Khare et al. found that in both standard and CRC patient samples, the proteins p68, β-catenin, and RELA exhibit a strong positive connection [70].

Both p68 and β-catenin elevated RELA mRNA and protein expression. RELA promoter activity was increased by p68, β-catenin, and Wnt (Figure 8D). p68 and β-catenin knockdown, on the other hand, decreased RELA promoter activity and resulted in lower RELA mRNA and protein expression. p68 was thought to occupy the RELA promoter with β-catenin at the TCF4/LEF binding element (TBE) sites, resulting in RELA transcription being potentiated. The p68 and β-catenin alliance positively regulated the NF-κB target genes. Findings in clinical samples confirmed that p68 increased NF-κB target gene expression. Tumors stably expressing p68 in a mouse transplant model confirmed the in vitro findings. This novel mechanism explains how p68 and β-catenin work together to regulate RELA expression and stimulate the NF-κB signaling axis to promote colon carcinogenesis. This mechanism proposes a potential therapeutic target in CRC by inhibiting NF-κB [52,53]. Integrative proteogenomic profiling appears to have revealed novel therapeutic opportunities for targeting signaling proteins in colon cancer treatment. This unique theory could pave the way for significant progress in molecularly driven precision treatment for colon cancer [71].

## 5. Conclusions

The results from the 12 research papers short-listed in this scoping review suggest that both γT3 and δT3 have potent anticancer effects and these T3 analogues exert anticancer effects through three major pathways, i.e., apoptosis (BIRC3, BIRC5, CASP8, CASP9, and PARP1), transcriptional dysregulation in cancer (CDKN1A, CDKN1B, MMP9, MYC, JUN, and RELA), and cancer progression (CASP3, CCND1, CTNNB1, VEGFA, and WNT1) pathways. Tocotrienol research has made significant progress in the last decade. It is evident that T3s, in particular γT3 and δT3, are promising anticancer agents for CRC. The World Cancer Research Fund and the American Institute for Cancer Research agreed that further efforts are needed to implement vitamin E as a preventive treatment for CRC. To date, no human trials are using γT3 or δT3 as anticancer agents against CRC due to insufficient data to support this.

## Figures and Tables

**Table 1:** Data extracted from the 12 cell-based research studies.

Study	Year	CRC Cell Lines	T3s	Protein(s) and Gene(s) Modulated		Main Outcomes	
Shibata et al. [16]	2010	DLD-1	δT3	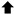	CDK-p21(*Cdkn1A);* CDK-p72 (*Cdkn1b);* GADD45 alpha (*GADD45A)*; Caspase-3 (*CASP3);* Caspase-7 (*CASP7);* Caspase-9 (*CASP9)*; *APAF1; elegans CED-4*	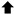	Cell cycle arrest; Apoptosis signals
Yang et al. [17]	2010	HT-29 HCT-116	δT3 or γT3	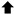	PARP-1	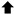	Cell cycle arrest; Apoptosis (Cleavage; PARP activation and DNA fragmentation)
				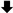	RhoA; HMGCR	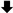	G-protein geranylgeranylation.
Kannappan et al. [18]	2010	HCT-116 HT-29	γT3	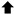	TRAIL; ERKs; Caspase-3; Caspase-8; Caspase-9; PARP-1	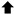	Cell death; apoptosis, DR4 and DR5; ROS
				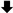	cIAP2; Bcl-xL; p38 MAPK	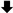	Cell survival proteins
Zhang et al. [19]	2011	SW620	δT3	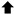	c-myc	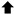	Swelling of mitochondria/ER; paraptosis-like cell death
				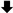	β-catenin; Wnt-1; Cyclin D1; c-Jun; MMP-7	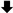	Cell viability
Xu et al. [20]	2012	HT-29	γT3	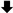	Wnt (No specification); β-catenin; TCF; Survivin; Cyclin D1; *Myc*	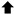	Shrunk/floated cells; apoptotic changes; apoptosis
						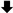	Adhesive ability; cell proliferation
Zhang et al. [21]	2013	SW620 HCT-8	δT3	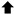	Caspase-3	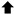	Cell size; round cells; paraptosis-based cell death; cytoplasmic vacuolization
				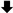	β-catenin; Cyclin D1; c-Jun; Wnt-1	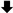	Proliferation
Shibata et al. [22]	2015	DLD-1	δT3	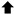	Caspase-3; Caspase-9; CDK-p21 *(Cdkn1a);* CDK-p72 *(Cdkn1b)*	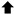	Apoptosis; cell cycle arrest; hypoxia genes/proteins expression > Normoxia genes/proteins
				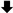	Akt–Thr^308^ and Ser^473^; CDK4; HIF-1α	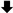	Cell proliferation
Yusof et al. [23]	2015	HT-29 SW837	γT3		No significant effect	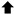	Distortion and shrinkage of cells; pyknosis and apoptotic bodies, chemoprevention
						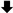	Proliferation
Abubakar et al. [24]	2016	HT-29	δT3	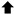	Caspase-3; Caspase-8	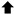	Apoptosis
				~	Caspase-9		
Eitsuka et al. [25]	2016	DLD-1	δT3	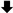	*hTERT*	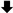	Cellular telomerase activity; Proliferation
Prasad et al. [26]	2016	HCT-116 HT-29 Caco-2	γT3	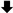	Survivin; cIAP1; cIAP2; Cyclin D1; c-Myc; MMP-9; CXCR-4; VEGF; ICAM-1; NF-κB/p65	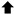	Apoptosis; cell cycle arrest
						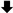	Proliferation; colony formation; expression of tumorigenic and metastasis proteins
Husain et al. [27]	2019	HCT-116 HT-29 SW480 SW620	δT3	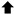	PARP1; Phosphatidylserine	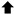	Apoptosis; cell cycle arrest
				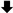	E-cadherin; Vimentin; MMP-9; VEGF; NF-κB/p65; β-catenin	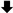	Colony formation; EMT; angiogenesis; migration; invasion and metastasis

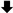
 Downregulat.ed; 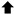 Upregulated; **~** Uncertain; δT3: delta-tocotrienol; γT3: gamma-tocotrienol; IC50: half-maximum inhibitory concentration; CASP: caspase; DR: death receptors; EMT: epithelial-mesenchymal transition; ER: endoplasmic reticulum; hr: hour; PARP: poly (ADP-ribose) polymerase; ROS: reactive oxygen species; T3s: tocotrienols.

**Table 2:** Summary of clinically relevant genes and proteins modulated by tocotrienols in human colon cancer cell lines.

Pathways Involved	CBs Modulation by T3s	Reported Effects in Colon Cancer Patients	Ref.
Apoptosis	*Caspase 3* ( 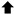 )	● Irradiated CRC cells from patients with lower levels of caspase-3 was associated with poor prognosis	[45]
	*Caspase 8* ( 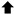 )	● Higher prevalence of mutations in the *caspase-8* genes in invasive carcinomas; reduce apoptotic activity	[46,47]
	*Caspase 9* ( 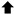 )	● Expression of the *caspase-9* gene downregulated in CRC tissue compared to surrounding normal mucosa	[48]
Transcriptional dysregulation in cancer	*CDKN1A (p21)* ( 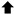 )	● P21 was downregulated in 50% (371/737) of CRC samples	[49]
	Jun family ( 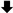 )	● Higher c-Jun expression observed in human colorectal adenocarcinomas	[50]
	*c-MYC* ( 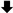 )	● CRC patients with higher MYC expression significantly shorter progression-free survival time and overall survival	[51]
	*RELA (NF-kB/p65)* ( 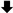 )	● Reduced RELA expression, resulting in deceased activation of the NF-κB signaling pathway, which inhibited carcinogenesis	[52,53]
Cancer progression	*CCND1* ( 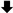 )	● CCND1 gene was detected in tumors from about 50% (54 out of 111) of CRC patients; absent in normal mucosa	[54]
	*VEGFA* ( 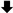 )	● Elevated expression of the VEGF family, especially of VEGFA, was reported in CRC patients with LNM	[55]
	*CTNNB1* ( 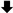 )	● CTNNB1 codes for β-catenin, which supports tumor growth ● A significant link between mutations in CTNNB1 gene and MSI	[56]

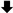
 Downregulat.ed; 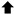 Upregulated; CB: candidate biomarkers; CRC: colorectal carcinoma; LNM: lymph node metastasis; MSI: microsatellite instability; T3s: tocotrienols. Italics refer to the gene name of the protein.

## Supplementary Materials

The following are available online at https://www.mdpi.com/article/10.3390/nu13114056/s1. Table S1: Inclusion and exclusion criteria for screening and full-text review.

## Abbreviations

Akt	protein kinase B
APAF1	apoptotic protease activating factor 1
APC	adenomatous polyposis coli
BAX	BCL2-Associated X Protein
Bcl-xL	B-cell lymphoma-extra large
BCL2	B-cell Lymphoma 2
BRAF	proto-oncogene, serine/threonine kinase
c-Jun	avian Sarma virus-17 oncogene (Jun comes from Japanese JU-NANA)
c-Myc	cellular homologue of avian myelocytomatosis virus
CASP3	caspease-3
CASP8	caspease-8
CASP9	caspease-9
CB	candidate biomarker
CC3	Cleaved Caspase-3
CDK-p21	Cyclin-Dependent Kinase Inhibitor 1
CDK-p72	Cyclin-Dependent Kinase Inhibitor 1B
CDK4	cyclin-dependent kinase 4
CI	combinational index
cIAP1	cellular inhibitor of apoptosis protein-1
cIAP2	Baculoviral IAP Repeat-Containing Protein 3
CIN	Chromosomal Instability
COX-2	Cyclooxygenase 2
CRC	colorectal carcinoma
CRISPR	clusters of regularly interspaced short palindromic repeats
CXCR-4	C-X-C chemokine receptor type 4
Cyt *c*	cytochrome complex
DCC	deleted in colorectal carcinoma
DR	death receptor
EGFR	epidermal growth factor receptor
EMT	epithelial–mesenchymal transition
ERKs	extracellular signal-regulated kinases
FAP	Familial Adenomatous Polyposis
FCC	Familial Colorectal Cancer
FOLFIRI	folinic acid, fluorouracil and irinotecan
GADD45	Growth Arrest and DNA Damage-inducible
GDT	Gamma-Delta Tocotrienol
HDI	Human Development Index
HIF-1α	hypoxia-inducible factor 1-alpha
HME	High MYC Expression
HMGCR	HMG-CoA reductase
HNPCC	Hereditary Non-Polyposis Colorectal Cancer
hTERT	human telomerase reverse transcriptase
IC_50_	half maximum inhibitory concentration
ICAM-1	intercellular adhesion molecule 1
IL-8	interleukin-8
KEGG	Kyoto Encyclopedia of Genes and Genomes
KO	Knockout
KRAS	Kirsten rat sarcoma viral oncogene homolog
LEF	lymphoid enhancer-binding factor
LME	low MYC Expression
LNM	lymph node metastasis
MCRC	metastatic colorectal cancer
miRNAs	micro-ribonucleic acids
MLH1	MutL homolog 1
MMP-7	Matrix metalloproteinase-7 or Matrilysin
MMP-9	Matrix Metallopeptidase 9
MSH2	MutS homolog 2
MSI	Microsatellite Instability
MSI-H	Microsatellite Instability High-Frequency
MSI-L	Microsatellite Instability Low-Frequency
NF-κB	nuclear factor kappa-light-chain-enhancer of activated B cells
OS	overall survival
*p*-value	calculated probability
p38 MAPK	p38 mitogen-activated protein kinases
p53	tumor suppressor protein (MW = 53 kD)
PARP-1	poly [ADP-Ribose] polymerase 1
PFS	progression-free survival
PKC	protein kinase C
PPI	protein-protein interaction
PRISMA	preferred reporting items for systematic reviews and meta-analyses
RhoA	transforming protein
Ser^473^	serine kinase
SMAD4	such as mothers against decapentaplegic homolog 4
T3s	tocotrienols
TBE	TCF4/LEF Binding Elements
TCF	T-cell factor
TCF4	Transcription Factor 4
TGF-RII	transforming growth factor receptor II
Thr^308^	threonine kinase
TRAIL	tumor Necrosis Factor-Related Apoptosis-Inducing Ligand
TRF	tocotrienol-rich fraction
TSG	tumor suppressor genes
TT	target therapy
VEGF	vascular endothelial growth factor
VEGFA	vascular endothelial growth factor A
Wnt	Wingless-related integration site
Wnt-1	Wingless-related integration site-1
αToc	alpha-tocopherol
γT3	gamma-tocotrienol
δT3	delta-tocotrienol
