# Supplementary material for: Clinically Relevant Genes and Proteins Modulated by Tocotrienols in Human Colon Cancer Cell Lines: Systematic Scoping Review
Source: Nutrients. 2021 Nov 12;13(11):4056. doi: 10.3390/nu13114056 (PMC8625890; doi:10.3390/nu13114056)
Supplement: Supplementary file 1 [file nutrients-13-04056-s001.zip › Supplementary Material (1).pdf]

PICO Worksheet and Search Strategy Protocol

1. Defining PICO question by identifying: Patient/Problem (P), Intervention (I), Comparison (C), and Outcome (O):
   - ☑ Patient/Problem: **Human colorectal cancer cells**
   - ☑ Intervention: **Tocotrienols (α, β, γ, & δ)**
   - ☑ Comparison: **Untreated colorectal cancer cells *(if any)***
   - ☑ Outcome: **Modulated genes and proteins**

2. Type of PICO question:
   - ☑ **Therapy**
   - ☑ **Prevention**
   - ☐ Diagnosis
   - ☐ Etiology
   - ☐ Prognosis

3. The main topics and alternate terms that can be used for the search:
   - ☑ **Tocotrienols**
   - ☑ **Genes**
   - ☑ **Proteins**
   - ☑ **Colorectal cancer**
   - ☑ **Colon cancer**

∴ PICO question: What genes and proteins that tocotrienols modulate in colorectal cancer cells to induce anti-cancer effects?

4. Type of studies to include in the search:
   - ☑ **Research studies or articles**
   - ☐ Meta-analysis
   - ☐ Systematic review
   - ☐ Clinical practice guidelines
   - ☐ Randomized controlled trial
   - ☐ Case report or series
   - ☐ Research report or other grey literature

5. Additional limits that may apply to the search:
   - ☑ **Year(s) of publication: 2010-2021 (Present)**
   - ☑ **Language(s): English**
   - ☐ Gender: N/A
   - ☐ Age: N/A

6. The databases for the search:
   - ☑ **Ovid MEDLINE**
   - ☑ **PubMed**
   - ☑ **Scopus**
   - ☑ **Embase**

7. PICO search strategy:
   - Tocotrienol* [Truncated – Stemming]
   - **AND** Gene* [Truncated – Stemming] **OR** Protein* [Truncated – Stemming]
   - **AND** Colorectal **OR** Colon
   - **AND** Cancer

∴ **Table S1.** Inclusion and exclusion criteria for screening and full-text review.

Category	Included	Excluded
Problem type	Human colon cancer cells	Animal colon cancer cells
Intervention type	Tocotrienols (α, β, γ, & δ)	Tocopherols (α, β, γ, & δ)
Outcome type	Modulated genes/proteins	Synergistic effects
Study type	Research studies only	All other types of studies and reviews
Study design	*In vitro* studies only	*In vivo* studies and others
Publication Language	English only	Any other languages
Publication period	Published between 2010-2021	Published before 2010
